# *Simplicillium sinense* sp. nov., a novel potential pathogen of tinea faciei

**DOI:** 10.3389/fmicb.2023.1156027

**Published:** 2023-05-12

**Authors:** Qiu-Hong Yan, Qing-Rong Ni, Wei-Jie Gu, Hong-Wei Liu, Xiao-Ying Yuan, Jing-Zu Sun

**Affiliations:** ^1^Medical Mycology Center, Department of Dermatology, Air Force Medical Center, Fourth Military Medical University, Beijing, China; ^2^State Key Laboratory of Mycology, Institute of Microbiology, Chinese Academy of Sciences, Beijing, China; ^3^Savaid Medical School, University of Chinese Academy of Sciences, Beijing, China

**Keywords:** pathogen, *Simplicillium sinense*, phylogenetic analyses, tinea faciei, antifungal susceptibility

## Abstract

*Simplicillium* species are widely distributed with a broad spectrum of hosts and substrates. Generally, these species are entomopathogenic or mycoparasitic. Notably, some isolates of *Simplicillium lanosoniveum* and *Simplicillium obclavatum* were obtained from human tissues. In this study, two fungi were isolated from the annular itchy patch of infected skin of a 46-year-old man with diabetes mellitus. Based on a combination of morphological characteristics and phylogenetic analysis, a novel species, *Simplicillium sinense*, was introduced herein. It morphologically differs from the remaining *Simplicillium* in the size of phialides and conidia. Additionally, it grows slowly on YPD at 37°C. Antimicrobial susceptibility testing presented that this fungus is resistant to most azole antifungals. Therefore, the diagnosis of tinea faciei was made, and after 2 weeks of being treated with oral terbinafine (250 mg, once a day) and topical terbinafine cream for 1 month, the rash was mainly resolved and no recurrence happened after 6 months of follow-up. Herein, *Simplicillium sinense* was introduced as a new fungal taxon. Meanwhile, a case of superficial infection caused by *S. sinense* was reported. So far, it is the third *Simplicillium* species obtained from human tissue. Meanwhile, terbinafine is recommended as the first-line antifungal treatment against *Simplicillium* infection.

## Introduction

Fungi have caused various infectious diseases in humans, ranging from superficial fungal infections to invasive fungal diseases, which affect approximately 15% of global population (Fisher et al., 2022). Among those fungi, *Acremonium* has drawn the attention of clinicians and mycologists, as a potential pathogen in patients with and without underlying risk factors (Birgit et al., 2008; Das et al., 2010; Perez-Cantero and Guarro, 2020). However, *Acremonium* is a large polyphyletic fungal genus that includes many *Acremonium-* and *Acremonium-*like fungi with similar morphological characteristics (Perdomo et al., 2011; Summerbell et al., 2011). Phylogenetic analysis has been supposed as an efficient approach in recognizing *Acremonium* and *Acremonium* allied fungi. It revealed that there are several clusters containing multiple *Acremonium* species including *Gliomastix*, *Sarcopodium*, *Sarocladium*, *Trichothecium*, and *Simplicillium* within the Hypocreales (Summerbell et al., 2011).

Based on phylogenetic analysis, *Simplicillium* W. Gams & Zare (Cordycipitaceae) was introduced for the accommodation of species previously placed in the *Verticillium* sect. *Prostrata* (Zare et al., 2000; Zare and Gams, 2001), which was mainly characterized by producing solitary phialides. Subsequently, more than 30 members of *Simplicillium* were introduced based on phylogenetic analysis (Wei et al., 2019; Chen et al., 2021, 2022). *Simplicillium* species are widely distributed and can be found in a broad spectrum of hosts and substrates, such as animals, insects, nematodes, plants, fungi, algae, soil, water, and even from human nails (Zare and Gams, 2001; Wei et al., 2019; Chen et al., 2021, 2022) For instance, *Simplicillium obclavatum* can produce multiple xylanases and endoglucanases, and this fungus was supposed to be used in biofuels and forage (Roy et al., 2013). However, *S. obclavatum* CBS 101713 was isolated from the human nail in Saudi Arabia (Zare and Gams, 2001), indicating a potential pathogenicity to humans.

During the investigation of causing agent of the face rash of a 46-year-old man, two fungal isolates were obtained from the pitches of his cheek skin. Based on a combination of morphological characteristics and phylogenetic analysis, a novel species, *Simplicillium sinense*, was introduced herein. Although the pathogenetic test was not carried out following the classical Koch’s postulates, *S. sinense* grows slowly on YPD at 37°C. Additionally, after 2 weeks of being treated with oral terbinafine (250 mg, once a day) and topical terbinafine for 1 month, the rash was mainly resolved and no recurrence happened after 6 months of follow-up. Therefore, the diagnosis of tinea faciei was made, and *Simplicillium sinense* was considered as a new potential agent causing this superficial fungal infection.

## Materials and methods

### Ethics statement

This study was performed in compliance with relevant laws and institutional guidelines and was approved by the Ethics Committee of Air Force Medical Center (2021-223-PJ01), and informed consent was obtained. The authors confirm the ethical policies of the journal, as noted on the journal’s author guidelines page.

### Case presentation

A 46-year-old man with diabetes found a pea-sized, round red itching rash on his left cheek 2 years ago. He visited the doctor in the clinic and was prescribed a topical application of mometasone furoate ointment. After the topical application of this drug for 10 days, the skin lesions subsided. Thereafter, the skin lesions recurred after repeated application of mometasone furoate ointment, and their boundary gradually expanded. He never had cats, dogs, and other pets, and his family members had no similar symptoms. Because of work needs, the patient often stayed up late and worked overtime. He had a 5-year history of diabetes mellitus. On examination, the dark red, annular scaly patch (approximately 5 cm × 6 cm) with lightly raised borders and central clearing was seen on the cheek of the patient’s left face. His skin lesions were further observed under an Olympus dermoscope.

### Mycology

Surface scrapings were obtained from the border of the skin lesions on the patient’s cheek for direct microscopic examination of potassium hydroxide (KOH). Surface scrapings were sterilized for 15 s with 75% alcohol and blood with sterilized water for 1 min and then inoculated on the Sabouraud medium. Finally, the subculture was incubated on yeast extract peptone dextrose agar (YPD) at 30 and 37°C, respectively, for 7 days. Our newly obtained isolate was deposited in the Air Force Medical Center Culture Collection (AFMCC) and was preserved in the China General Microbiological Culture Collection Center (CGMCC) as well. A Nikon Ellipse 80i light microscope, equipped with differential interference contrast (DIC) optics, was used to capture digital images. Tarosoft (R) v.0.9.7 Image Frame Work was used to measure the morphological structures and the Adobe Photoshop CS6 image 22 software (Adobe Systems, United States) to edit the photographic plates.

For observation by SEM, the fungus was inoculated on a PDA medium at 30°C. After 7 days, two patches (0.3 cm × 0.3 cm) of the fungal colony were fixed in 2.5% glutaraldehyde in 0.1 M phosphate-buffered saline (BPS, pH 7.2) at 4°C. Then, the samples were dehydrated and sputter-coated by following the method described by Sun et al. (2023). Samples were loaded on the SEM (SU8010, Hitachi, Tokyo, Japan) and observed and photographed.

### DNA extraction, PCR amplification, and sequencing

The genomic DNA of stains 16a and 16b was extracted from fresh mycelium growing on PDA after 10 days of growth following the rapid “thermolysis” method described in the previous study (Zhang et al., 2010). The ITS4/ITS5 primer pair is used for amplification of the internal transcribed spacer gene region (ITS; White et al., 1990), and the 983F/2218R primer pair is employed for amplification of the partial translation elongation factor 1-alpha gene region (TEF-1α; Carbone and Kohn, 1999). Each PCR reaction system followed the descriptions by Sun et al. (2023). The PCR products were sequenced by Beijing Tianyihuiyuan Bioscience and Technology.

### Phylogenetic analyses

SeqMan Pro v. 7.1.0 (DNASTAR Lasergene) was used to trim the low-quality bases at both ends of the raw forward and reverse reads and to assemble them. The newly obtained sequences were queried against the nuclear database of NCBI. A total of 72 taxa of *Simplicillium* and two outgroup taxa (*Purpureocillium lilacinum*) were cascaded for phylogenetic analysis. The alignments were generated by using MAFFT version 7.03 with the Q-INS-I strategy (Katoh and Standley, 2013). Conserved blocks were selected from the initial alignments with Gblocks 0.91 b (Castresana, 2000). The best nucleotide substitution model for each gene was determined by using jModelTest 2.1.1 (Darriba et al., 2012). GTR + G was estimated as the best-fit model for ITS and TEF1-α genes under the output strategy of BIC. The multi-locus phylogenetic analyses included 604 characters for ITS and 940 characters for TEF. All characters were weighted equally, and gaps were treated as missing characters.

Maximum likelihood (ML) analyses were performed by RAxML2.0 (Edler et al., 2021), using the GTR-GAMM model. The maximum likelihood bootstrap proportions (MLBP) were using 1,000 replicates. Bayesian inference (BI) analyses were conducted with MrBayes v3.2.7 (Ronquist et al., 2012). Metropolis-coupled Markov Chain Monte Carlo (MCMC) searches were calculated for 10,000,000 generations, sampling every 100th generation with the best best-fit model for each gene. A total of two independent analyses with six chains each (one cold and five heated) were carried out until the average standard deviation of the split frequencies dropped below 0.01. The initial 25% of the generations of MCMC sampling were discarded as burn-in. The refinement of the phylogenetic tree was used for estimating Bayesian inference posterior probability (PP) values. The tree was viewed in FigTree v1.4 (Rambaut, 2012), with values of maximum likelihood bootstrap proportions (MLBP) greater than 50% and Bayesian inference posterior probabilities (BIPP) greater than 95% at the nodes, which are shown along branches.

### Antifungal susceptibility

Antifungal susceptibility was carried out by following the protocol (M38, reference method for broth dilution antifungal susceptibility testing of filamentous fungi, a standard for global application developed through the Clinical and Laboratory Standards Institute consensus process) with a little bit of modification. The isolate 16a was inoculated on a YPD medium at 30°C for 10 days, and then, the conidia were harvested by flooding with approximately 1 mL sterile 0.85% saline. Conidia were resuspended by gentle probing. The conidial suspension was adjusted to an absorbance of 0.13 at 530 nm. The conidia were diluted with YPD liquid medium in a ratio of 1:50 conidial suspension vs. YPD and inoculated each well with 0.1 mL of the corresponding antibiotic. The wells were cultivated at 30°C for 72 h and read the absorbance at 530 nm.

## Results

### Clinical manifestations, mycology, and treatment outcome

**Figure 1:**
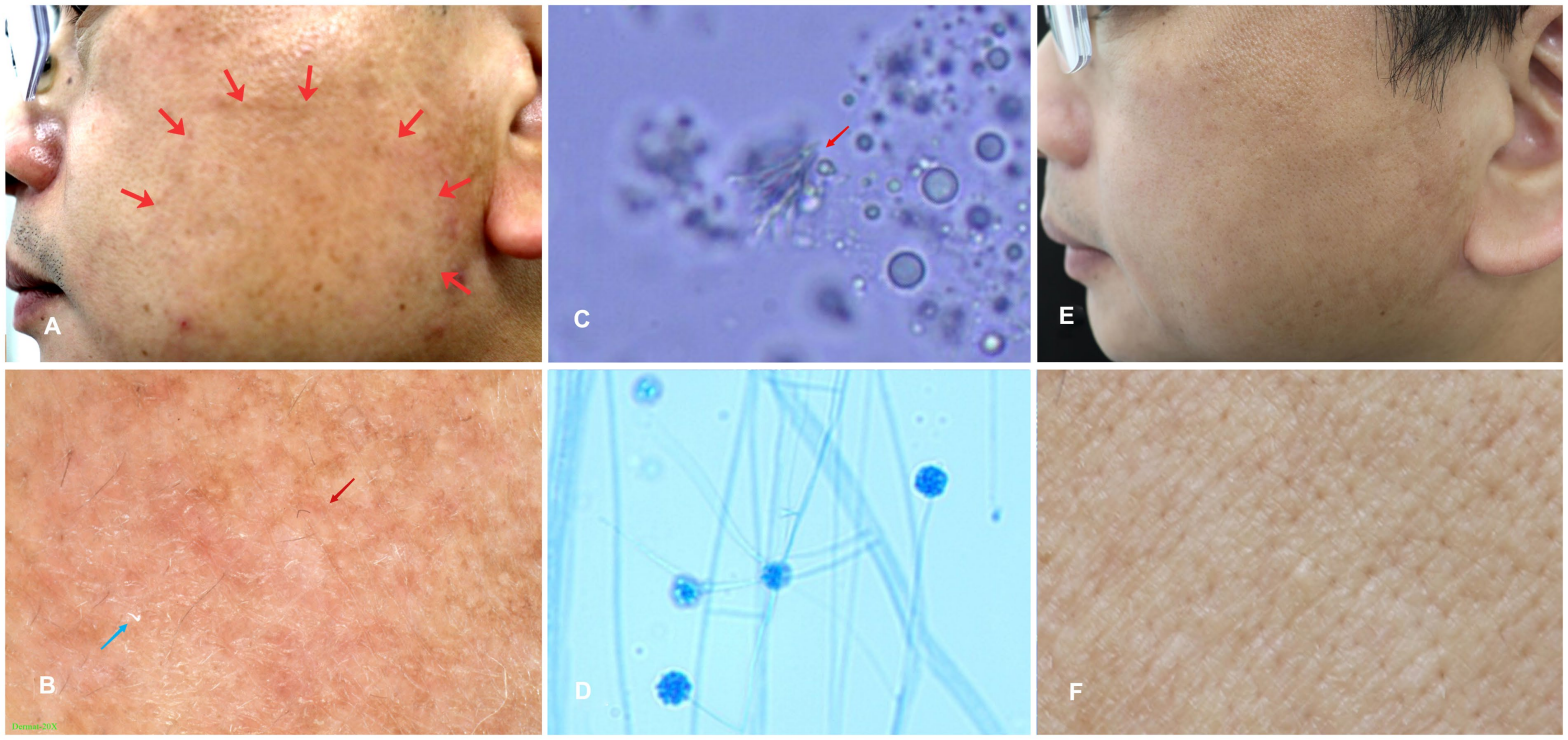
**(A)** Dark red, annular scaly patch (approximately 5 cm × 6 cm) with lightly raised borders and central clearing was seen on the cheek of the patient’s left face; **(B)** Dermoscopic examination of the skin lesion showed diffuse erythema (red asterisks) and white peripheral scales (blue asterisks); **(C)** The hyphae from skin scrapings; **(D)** Microscopic examination of small culture showed that the spores were agglomerated at the top of hyphae like raspberry; and **(E,F)** The rashes were mainly resolved after being treated with oral terbinafine (250 mg, once a day) for 2 weeks and topical terbinafine for 1 month.

A 46-year-old man presented with a 2-year history of an itchy rash on his left face (Figure 1A). Physical examination showed a dark red, annular scaly patch (approximately 5 cm × 6 cm) with lightly raised borders and central clearing on the left cheek of the patient’s left face (Figure 1A). Dermoscopic analysis of the patient’s lesion showed diffuse erythema and white peripheral scales (Figure 1B). Direct microscopic examination showed the thin fungal hyphae from the scraping of the lesions using potassium hydroxide (KOH; Figure 1C). After 2 weeks of being treated with oral terbinafine (250 mg, once a day) and topical terbinafine cream for 1 month, the rash was mainly resolved (Figures 1E,F).

### Phylogenetic analyses

**Figure 2:**
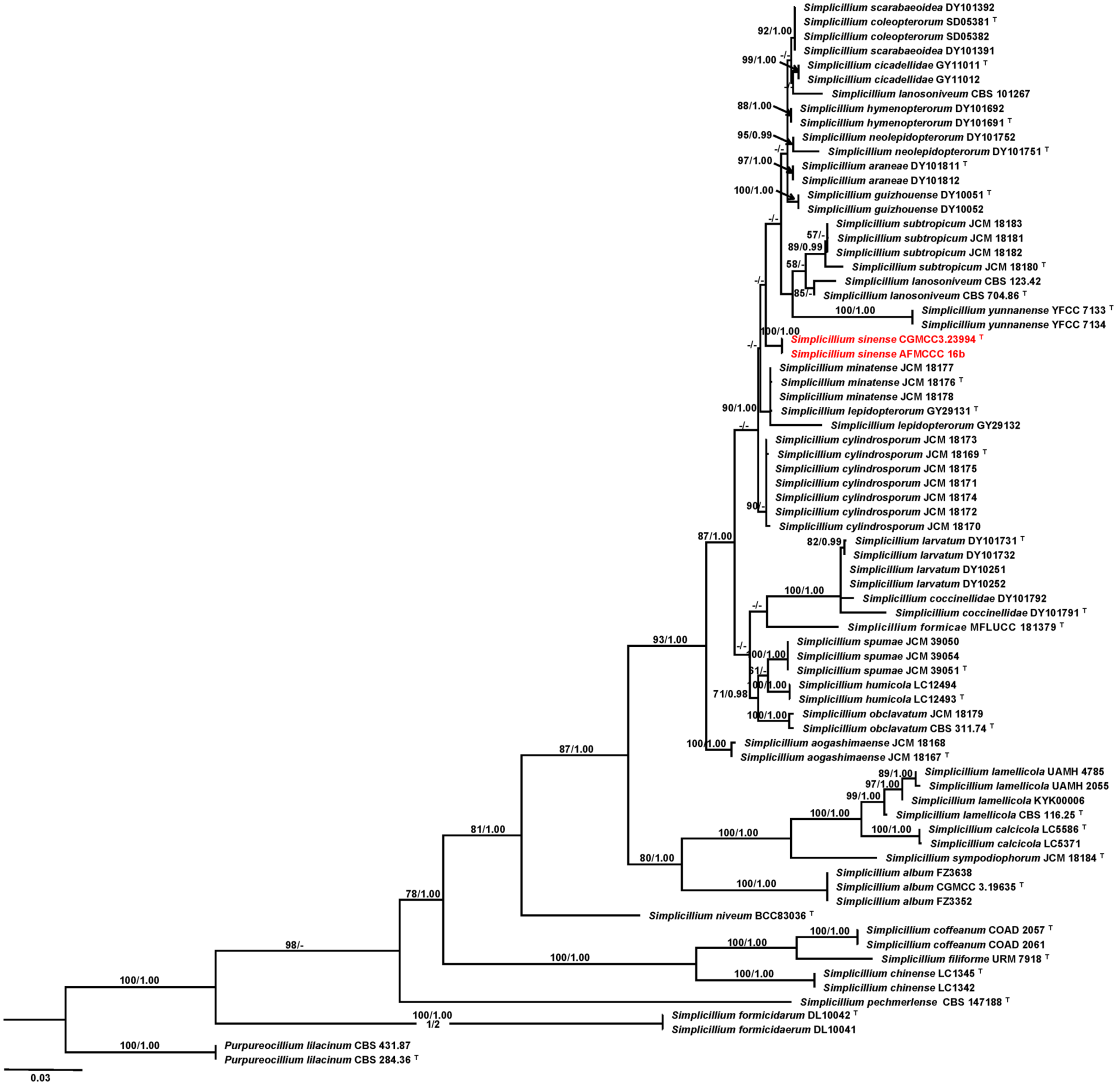
Phylogenetic tree based on maximum likelihood analysis of the combined ITS and TEF1-a dataset. Two taxa of *Purpureocillium lilacinum* are the outgroup taxa. Bootstrap values higher than 50% from RAxML (MPBP; left) and Bayesian posterior probabilities greater than 0.95 (BIPP; right) are given above the nodes. Dashes indicate bootstrap values of less than 50% or Bayesian posterior probabilities lower than 0.95.^T^ indicates ex-type isolates; isolates obtained in this study are in red.

The multi-gene phylogenetic analysis based on a cascade dataset of ITS and TEF-1α presented that our isolates AFMCCC16a and AFMCCC 16b were positioned within *Simplicillium*, which formed a distinct terminal that differs from the remaining *Simplicillium* (MLBP = 100, BIPP = 1.00). Phylogenetically, it grouped with *Simplicillium araneae*, *S*. *cicadellidae*, *S*. *coleopterorum*, *S*. *guizhouense*, *S*. *humicola*, *S*. *hymenopterorum*, *S*. *neolepidopterorum*, *S*. *subtropicum*, *S. lanosoniveum*, *S*. *scarabaeoidea*, and *S*. *yunnanense* but not well supported (Figure 2).

### Taxonomy

**Figure 3:**
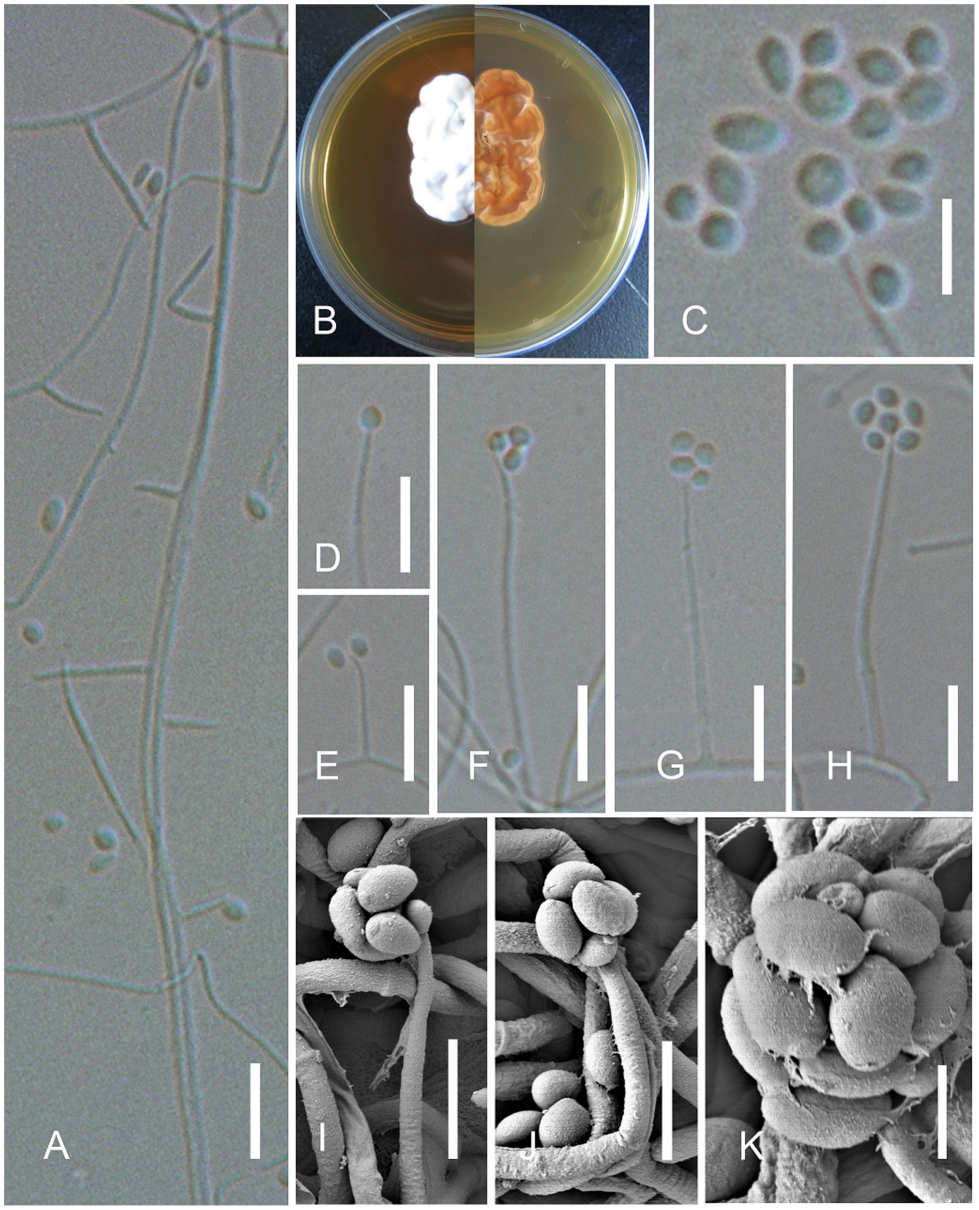
*Simplicillium sinense* X.Y. Yuan, Jing Z. Sun & H.W. Liu, sp. nov. (sympodia). **(A)** Conidiophores; **(B)** Cultures on YPD medium from the surface and reverse; **(D–K)** Phialides and conidia; **(C)** Conidia. Scale bars: **A,D–H** = 10 μm; **C,I,J** = 5 μm; and **K** = 2 μm.

***Simplicillium sinense*** X.Y. Yuan, Jing Z. Sun & H.W. Liu, sp. nov. (Figure 3).

**MycoBank number:** MB847412.

**Etymology:** the epithet “sinense” refers to the discovery locality China of this species.

**Type:** AFMCCC 16a, CGMCC3.23994.

**Description**: On the skin of humans. ***Asexual morph***: Hyphomycetous. *Mycelium* aseptate, or less septate, hyaline, smooth-walled. ***Phialides*** 10–30 × 0.8–1.5 (x̄ = 26 × 1.0, *n* = 30) μm, arising from the prostrate mycelium, blastic, enteroblastic, phialidic, monophialidic, discrete, terminal, aseptate or 1-septate, hyaline, smooth-walled, solitary, tapering toward the apex. ***Conidia*** 1.2–2.0 × 1.0–2.0 (x̄ = 1.8 × 1.5, *n* = 50) μm, hyaline, amerospores, globose to ellipsoidal, smooth-walled, adhering in globose to ellipsoidal head at the apex of phialides. ***Sexual morph***: Undetermined.

**Culture characters:** The colonies on YPD medium were slow-growing, reaching a diameter of 2.5 cm in 10 days at 30°C, growing slowly at 37°C, white, entire margin, velvety, with radial cracks and primrose-yellow on the reverse.

**Material examined:** CHINA, Beijing, Air Force Medical Center, on a 46-year-old man’s cheek, 19 February 2021, Qiu-Hong Yan (AFMH16 holotype; living culture: AFMCCC 16a, CGMCC3.23994). Sequences generated from this strain have been deposited in GenBank with accession numbers as follows: ITS (OQ332403) and TEF-1α (OQ352167).

**Additional material examined:** CHINA, Beijing, Air Force Medical Center, on a 46-year-old man cheek, 19 February 2021, Qiu Hong Yan (living culture: AFMCCC 16b). Sequences generated from this strain have been deposited in GenBank with accession numbers as follows: ITS (OQ332404) and TEF-1α (OQ352168).

**Teleomorph:** Undetermined.

**Notes:** Our isolates of *Simplicillium sinense* (AFMCCC 16a and AFMCCC 16b) colonized on the skin of humans (Figure 1). Phylogenetically, it grouped with *Simplicillium araneae*, *S*. *cicadellidae*, *S*. *coleopterorum*, *S*. *guizhouense*, *S*. *humicola*, *S*. *hymenopterorum*, *S. lanosoniveum*, *S*. *neolepidopterorum*, *S*. *scarabaeoidea*, *S*. *subtropicum*, and *S*. *yunnanense* but with week support value (Figure 2). When comparing the ITS sequence of *S. sinense* (AFMCCC 16a) with the aforementioned species, it presented 99% (5 bp differences out of 513) similarities against that of the type strain of *S*. *guizhouense* (DY10051), which is higher than that of other species, but when comparing the TEF-1α region sequence with those species, it showed 97.5% (26 bp differences out of 947) against that of *S*. *guizhouense* (DY10051). Morphologically, *S. sinense* differs from *S. guizhouense* in producing slender and short phialides (10–30 × 0.8–1.5 vs. 21.1–52.2 × 1.0–1.8 μm) and smaller conidia (1.2–2.0 × 1.0–2.0 vs. 2.4–2.9 × 1.6–1.8 μm; Chen et al., 2022). *Simplicillium sinense* presented a high similarity to *S. minatense* in shape and size of phialides [11–31(−47) × 1.0–1.7 μm] but the latter with larger conidia (2.0–3.5 × 1.8–2.5 μm) that of *S. sinense* (Nonaka et al., 2013; Wei et al., 2019). When compared with *S. minatense*, the ITS regions and TEF-1α of *S. sinense* (AFMCCC 16a) presented 98% (10 bp differences out of 552) and 99% (9 bp differences out of 922) similarities against that of the type strain of *S. minatense* (JCM 18176), respectively. Thereby, *S. sinense* was introduced as novel species according to the species boundary suggested by Vu et al. (2019).

### Antifungal susceptibility

The *in vitro* susceptibility testing results of the isolate are shown in Table 1, with corresponding minimum inhibitory concentration (MIC) of fluconazole (>256 μg/mL), itraconazole (>16 μg/mL), terbinafine (<0.03 μg/mL), voriconazole (0.25 μg/mL), posaconazole (0.5 μg/mL), isavuconazole (<0.03 μg/mL), and so on. The antifungal tests presented that *Simplicillium sinense* was resistant to fluconazole, itraconazole, caspofungin, 5-iodouracil, micafungin, anidulafungin, and anidulafungin but sensitive to terbinafine and isavuconazole.

**Table 1:** The antifungal susceptibility of *Simplicillium sinense.*

Antibiotics	MIC (μg/mL)	Result	Antibiotics	MIC (μg/mL)	Result
Fluconazole	>256	R	Micafungin	>8	R
Itraconazole	>16	R	Terbinafine	<0.03	S
Caspofungin	>8	R	Anidulafungin	>8	R
Amphotericin B	1		Posaconazole	0.5	R
5-Iodouracil	>64	R	Isavuconazole	<0.03	S
Voriconazole	0.25				

MIC, minimum inhibitory concentration.

## Discussion

Tinea faciei is a relatively uncommon superficial fungal infection limited to the glabrous skin of the face. It is mostly seen in pediatric patients, usually caused by dermatophytes (Lin et al., 2004; Kimura et al., 2015). *Acremonium* species are saprophytic molds that are ubiquitous in the environment and typically found in soil and potted plants. They are commonly encountered as contaminants and occasionally cause fungal infections such as onychomycosis or mycotic keratitis in both immunocompetent and immunocompromised patients (Gupta and Elewski, 1996; Read et al., 2000; Jacobs et al., 2020). Tinea faciei caused by non-dermatophyte molds (NDMs) such as *Acremonium* species have not been reported.

The genus *Simplicillium* was included in *Acremonium* species and segregated from the previous *Verticillium* sect. *Prostrata* in addition to *Haptocillium*, *Lecanicillium*, *Pochonia*, and *Rotiferophthora* (Zare et al., 2000; Sung et al., 2001; Zare and Gams, 2001). These species resemble *Lecanicillium* in total morphology, but they mainly produce solitary phialides (Wei et al., 2019; Chen et al., 2022). Because of the verticillate phialides, *Simplicillium lanosoniveum* and *Simplicillium obclavatum* were previously named *Acremonium lanosoniveum* and *Acremonium obclavatum* (Summerbell et al., 2011; Gams, 2017). The ITS gene region performed well in determining *Simplicillium* (Zare et al., 2000; Nonaka et al., 2013; Wei et al., 2019). The translation elongation factor 1-alpha gene region (TEF-1α) was recommended as a secondary barcode for fungi (Mirhendi et al., 2015; Meyer et al., 2019; Pakshir et al., 2020), which also performed well in species identification, especially in the recognition of hypocrealean fungi (Wei et al., 2019; Chen et al., 2021; Sun et al., 2023). In this study, our phylogenetic analysis based on ITS and TEF-1α also demonstrates well the interspecific relationship of *Simplicillium*. The isolates obtained from the patient formed a distinct terminal, representing a novel *Simplicillium* species.

*Simplicillium* species are widely distributed with a broad spectrum of hosts and substrates (Zare and Gams, 2001; Nonaka et al., 2013; Wei et al., 2019; Chen et al., 2021). Generally, these species are entomopathogenic or mycoparasitic (Wei et al., 2019; Chen et al., 2021, 2022). *Simplicillium lanosoniveum* was originally isolated from the *Cibotium schiedei* in a greenhouse, which was frequently isolated from the rust fungi *Hemileia vastatrix* and also from the pupa of *Pseudaulacaspis pentagona* (Zare and Gams, 2001; Chen et al., 2021). Despite it frequently acting as a parasite of plants, fungi, and insects, it was recently obtained from the hair of a giant panda and the bronchoalveolar lavage fluid of a patient, indicating a mammalian pathogenicity (Wei et al., 2019). *Simplicillium obclavatum* was originally isolated from the air above the sugarcane field, which was also isolated from the rust fungi on *Arachis hypogaea* (Zare and Gams, 2001). Notably, *S. obclavatum* CBS 101713 was isolated from the human nail in Saudi Arabia (Zare and Gams, 2001). *Simplicillium sinense* introduced herein was associated with human tissue. Based on our patient’s clinical manifestation, dermoscopic examination, mycology results such as direct microscopic examination and culture, and therapeutic response, *S. sinense* was reported as the potential pathogen causing tinea facie in this case. Although the pathogenetic test was not carried out following the classical Koch’s postulates, after 2 weeks of being treated with oral terbinafine and topical terbinafine for 1 month according to the antifungal susceptibility test, the rash was resolved and no recurrence happened after 6 months of follow-up. Therefore, the diagnosis of tinea faciei was made and *Simplicillium sinense* was considered as a new potential agent causing this superficial fungal infection. Species of *Simplicillium* are usually entomopathogenic and can digest the outercoat of insects due to the chitinase (Wei et al., 2019). The keratin of humans has similar chemical composts alike the outercoat of insects, which was thought of as a suitable substance for those fungi, especially in immunocompromised populations and certain diseases such as diabetes (Nair et al., 2023). In consideration of this case, diabetes mellitus may be a risk factor for this superficial fungal infection.

## Data availability statement

The data presented in the study can be found in NCBI and Mycobank repositories. The sequence accession number(s) can be found at: https://www.ncbi.nlm.nih.gov/genbank/, OQ332403, OQ332404, OQ352167, OQ352168, and the scientific name of the fungus can be found at https://www.mycobank.org/, MB847412.

## Ethics statement

The studies involving human participants were reviewed and approved by the Ethics Committee of Air Force Medical Center (2021-223-PJ01). The patients/participants provided their written informed consent to participate in this study. Written informed consent was obtained from the individual(s) for the publication of any potentially identifiable images or data included in this article.

## Author contributions

Q-HY and J-ZS mainly completed experiments and wrote the manuscript. Q-RN mainly contributed to writing the manuscript. X-YY designed the experiments and reviewed the manuscript. W-JG, H-WL, and J-ZS contributed to analyzing the clinical case and reviewing the manuscript. All authors contributed to the article and approved the submitted version.

## Funding

This research was jointly supported by the Military Key Project under Grant (20SWAQK12) and the National Key Research and Development Program of China (2021YFC2300400).

## Conflict of interest

The authors declare that the research was conducted in the absence of any commercial or financial relationships that could be construed as a potential conflict of interest.

## Publisher’s note

All claims expressed in this article are solely those of the authors and do not necessarily represent those of their affiliated organizations, or those of the publisher, the editors and the reviewers. Any product that may be evaluated in this article, or claim that may be made by its manufacturer, is not guaranteed or endorsed by the publisher.
